# Mediating Effect of Nursing Organizational Culture on the Relationship Between Ambidextrous Leadership and Staff Nurse Clinical Leadership

**DOI:** 10.1155/jonm/6625892

**Published:** 2025-04-11

**Authors:** Mengmeng Zeng, MengChao Zhang, Qiqi Ni, Yuezhong Wang, Xiaoyan Gong, Yiyu Zhuang

**Affiliations:** Nursing Department, Sir Run Run Shaw Hospital, Zhejiang University School of Medicine, Hangzhou, China

**Keywords:** ambidextrous leadership, mediating effect, nursing organizational culture, staff nurse clinical leadership, structural equation model

## Abstract

**Aim:** This study aims to explore the relationship of head nurses' ambidextrous leadership with staff nurse clinical leadership and the mediating effect of nursing organizational culture.

**Background:** Clinical nurses are an important part of the nursing team, and their clinical leadership is of great significance to improving the quality of nursing and developing a nursing career. The ambidextrous leadership of head nurses, which combines transformational and transactional leadership, is a new leadership style. There are few studies on the effect of head nurses' ambidextrous leadership on staff nurse clinical leadership.

**Methods:** This study was a cross-sectional study, and convenient sampling method was used to extract 500 nurses from first-class comprehensive hospital in Hangzhou City, Zhejiang Province, as research participants. The participants were administered an electronic questionnaire consisting of the General Information Questionnaire, Ambidextrous Leadership Style Scale, the Chinese version of Nursing Culture Assessment Tool, and the Chinese version of Clinical Leadership Survey. All the three scales used in this study had satisfactory construct validity, content validity, and reliability. Multiple stepwise linear regression analysis was used to explore the influencing factors of staff nurse clinical leadership. AMOS 24.0 software was used to construct a structural equation model to verify the mediating effect of nursing organizational culture between the ambidextrous leadership of head nurses and staff nurse clinical leadership.

**Results:** The score of staff nurse clinical leadership was 66.69 ± 7.42. Significant positive correlations were noted between staff nurse clinical leadership and head nurses' ambidextrous leadership (*r* = 0.461, *p* < 0.01), between nursing organizational culture and staff nurse clinical leadership (*r* = 0.685, *p* < 0.01), and between ambidextrous leadership and organizational culture (*r* = 0.641, *p* < 0.01). Professional title, role at work, marital status, participation in leadership training, and nursing organizational culture were the main predictors of staff nurse clinical leadership, collectively accounting for 49.8% of the total variation. Nursing organizational culture played a complete mediating role between head nurses' ambidextrous leadership and staff nurse clinical leadership, and the mediating effect was 0.555 (95% CI [0.454, 0.692]), accounting for 98.8% of the total effect.

**Conclusion:** Staff nurse clinical leadership represents the upper-middle level of clinical leadership. Nursing organizational culture can independently predict the level of staff nurse clinical leadership and has a complete mediating effect between head nurses' ambidextrous leadership and staff nurse clinical leadership. However, due to time, energy, and sampling methods, this study's sample is insufficient to represent the national nursing staff. Future research should further expand the survey's geographical scope and sample size.

**Implications for Nursing Management:** Senior managers can encourage the head nurses to adopt the ambidextrous leadership style and consequently raise the enthusiasm of the organizational culture to improve staff nurse clinical leadership. Managers should rework the rules and regulations and increase work enthusiasm among nurses. Furthermore, they should carry out hierarchical training of clinical leadership for nurses and reasonably empower them to increase their work autonomy and self-efficacy and to encourage and support them in their clinical leadership practice.

## 1. Introduction

With the increasingly severe problems associated with an aging population, changes in disease spectrum, and ecological disturbances in China, the country, society, and the people require medical and nursing services of higher quality. The World Health Organization emphasized the importance of developing nursing leadership in its report titled “The State of World Nursing 2020: Investing in Education, Employment, and Leadership” [1]. Currently, the world needs nurses more than ever to benefit from their knowledge, skills, and leadership needed to provide high-quality nursing care [1].

In recent years, scholars at home and abroad have believed that nursing leadership is not unique to head nurses and that every nurse may possess leadership skills and needs to develop them [2, 3]. Patrick et al. refer to the leadership possessed by clinical nurses who provide direct care to patients as staff nurse clinical leadership, which is defined as the behavior of nurses while providing guidance and support to patients and medical teams in the process of caring for patients [4]. The characteristics of this leadership behavior are rich clinical expertise, comprehensive understanding of others, and effective communication, coordination, and cooperation. Higher levels of clinical leadership among nurses can reportedly improve nursing quality and patient outcomes, promote teamwork, increase nurses' job satisfaction, strengthen nurses' professional identity, and reduce nurse turnover intention [5–8].

Clinical settings require frequent interactions between head nurses and nurses, with the closest form of communication being between head nurses and staff nurses. Head nurses' leadership is an important factor affecting staff nurses' attitudes and behaviors [9, 10]. Previous studies have shown that head nurses' leadership can affect staff nurse clinical leadership [4]. However, existing studies have mostly focused on exploring the impact of personal factors on staff nurse clinical leadership, such as personality traits [11], emotional intelligence [12], and cognitive competencies [13]. There is insufficient exploration of the influence of head nurses' leadership on staff nurse clinical leadership.

Ambidextrous leadership refers to the use of contradictory thinking by leaders, which allows them to make constant adjustments according to various organizational contexts, and it also involves being able to flexibly switch between the two leadership behaviors, thus eliminating conflicts between conflicting forces and possibly even exerting synergistic effects [14]. According to the conventional perspective, ambidextrous leadership can be divided into transactional leadership and transformational leadership [15]. Cai et al. found that head nurses' ambidextrous leadership (a combination of transformational leadership and transactional leadership) had a significant positive correlation with staff nurse clinical leadership [16]. Compared with a single leadership style, head nurses who adopt ambidextrous leadership, a combination of transformational and transactional leadership, can give staff nurses more power and resources to a certain extent; furthermore, they can better meet staff nurses' spiritual and material needs, stimulate their work enthusiasm, and encourage them to improve their own abilities. Furthermore, staff nurses can observe, imitate, and practice the leadership behavior of managers, thus helping and influencing other team members in their work [16]. However, research on leadership styles in the nursing field is mostly focused on a single leadership style, with the most attention paid to either the transformational style or the transactional style [17], and there is a lack of research on the impact of head nurses' ambidextrous leadership on staff nurse clinical leadership. However, there is a challenge in measuring leadership levels. Head nurses often perceive their leadership as relatively high, whereas clinical nurses are likely to underestimate head nurses' leadership level. In fact, head nurses' ambidextrous leadership should be focused on serving clinical nurses, and its effectiveness should be assessed based on the perception of clinical nurses. In this study, the scale used to measure leadership was completed by clinical nurses. This scale is easy to understand and objective, making it effective for assessing the level of head nurses' ambidextrous leadership as perceived by nurses.

Organizational culture is a shared set of values, assumptions, and beliefs that can guide and shape employees' attitudes and work behaviors [18]. Organizational culture helps individuals identify appropriate and inappropriate behaviors in an organization. Schein's organizational culture theory states that an organization's culture is largely influenced by the behavior of its leaders and that organizational culture determines organizational values and the behavior of organizational members under these values [19]. A positive nursing organizational culture can improve nurses' work enthusiasm [20], work engagement [21], work performance, and job satisfaction [22] while reducing the desire to leave [23, 24]. Furthermore, it can promote nurses' innovative behaviors [25], improve their core competencies [26], and improve the quality of nursing care provided [27]. Lee and Jang identified that negative organizational culture can increase nurses' work pressure and fatigue and subsequently increase turnover intention, which is not conducive to improving the quality of nursing service [28]. Similarly, de Vries and Curtis identified the lack of support from management and peers and a poor work environment as barriers to the development of staff nurse clinical leadership [29]. The nursing work environment is largely shaped by nursing organizational culture and is closely associated with the outcomes of patients and nurses [30]. The leadership style of managers markedly influences the formation and development of team culture [31]. However, the specific influence paths and causal relationships among head nurses' ambidextrous leadership, nursing organizational culture, and staff nurse clinical leadership remain unknown. There are many organizational culture measurement scales, and it is challenging to select one that is appropriate for this particular nursing organization. In this study, we selected the scale that was developed with nurses as the research object and later translated and culturally adapted by Chinese scholars. The scale can effectively measure the nursing organizational culture perceived by nurses in China and has been widely used in China.

Therefore, this study used Schein's organizational culture theory as the basis to explore the impact of head nurses' ambidextrous leadership and nursing organizational culture on staff nurse clinical leadership and verify the mediating effect of nursing organizational culture. Thus, it provided a reference for formulating intervention measures to improve staff nurse clinical leadership.

## 2. Methods

### 2.1. Study Design

This was a cross-sectional study conducted at the Sir Run Run Shaw Hospital, Zhejiang University School of Medicine, Hangzhou, China. This study was aimed at exploring the relationships among head nurses' ambidextrous leadership perceived by nurses, nursing organizational culture, and staff nurse clinical leadership. Convenience sampling method was used to select the research participants from the research institution. However, the convenience sampling method may lead to a lack of representation of the sample, making it difficult to generalize the findings to a larger scale.

The sample size calculation method for this study was related to the total number of items in the four scales, namely, the General Information Questionnaire, the Ambidextrous Leadership Style Scale (ALSS), the Chinese version of the Nursing Culture Assessment Tool (C-NCAT), and the Chinese version of Clinical Leadership Survey (C-CLS), and the sample size was at least 10 times the total number of items [32]. Given that the total number of items in this study was 46, the required minimum sample size was 460 cases. Assuming that sample loss and poor questionnaire completion quality could occur in 10% of the cases, we increased the target sample size by 10%, and the final calculated sample size was 506 cases. We distributed questionnaires to 506 nurses who met the inclusion and exclusion criteria of the study. After deleting low-quality questionnaires with regular or identical answers, a total of 472 valid responses were collected, with an effective recovery rate of 93.3%.

### 2.2. Inclusion and Exclusion Criteria

The inclusion criteria were as follows: (1) registered nurses with a Chinese nursing license, (2) in-service nurses who provided direct clinical care, and (3) nurses who volunteered to participate in this study and signed the informed consent.

The exclusion criteria were as follows: (1) informally employed nurses in hospitals, nurses employed in other hospitals, and nursing interns; (2) nurses who were not on duty during the survey period; and (3) nurses who had physical or mental illnesses and needed treatment.

### 2.3. Instruments

1. General information questionnaire. The scale was designed by the researchers and included 10 items, including gender, age, marital status, education, professional title, role at work, employment method, years of work experience, medical department of service, and participation in leadership training.
2. ALSS [33]. This scale comprises the transformational leadership style scale and the transactional leadership style scale. The ambidextrous leadership score is obtained by multiplying the average of the two subscale scores. A higher score indicates a higher opinion of the participants regarding their head nurses' ambidextrous leadership level. The transformational leadership style scale includes two dimensions: idealized influence and inspirational motivation. Similarly, the transactional leadership style scale includes two dimensions: contingent reward and active management by exception. The scale uses peer evaluation and the 5-point Likert scoring method, with points 1 and 5 representing strongly disagree to strongly agree, respectively. The feedback from the experts and respondents indicates that the scale has good content validity [33]. The average variance extracted (AVE) from the scale was above 0.5, with the square root of each variable's AVE exceeding its correlation coefficient, indicating good construct validity [33]. Cronbach's alpha coefficients of both the total scale and each subscale were greater than 0.7, indicating the scale's reliability [33]. In this study, the total Cronbach's alpha coefficient of the ambidextrous leadership scale was 0.896; Cronbach's alpha coefficients for the transformational leadership and transactional leadership scales were 0.927 and 0.781, respectively.
3. The C-NCAT [26]. Hu et al. translated and culturally adapted the C-NCAT [26]. The scale comprises 19 items across six dimensions, namely, behavior (*n* = 3), teamwork (*n* = 5), expectation (*n* = 3), communication (*n* = 3), satisfaction (*n* = 2), and professional commitment (*n* = 4), with one item shared between the behavior and teamwork dimensions. The scale uses self-assessment and the 4-point Likert scoring method. Higher scores indicate an increasingly positive nursing organizational culture atmosphere. The content validity of the C-NCAT was 0.968 [34]. The C-NCAT had satisfactory construct validity, with the AVE value ranging from 0.519 to 0.790 [26]. The total Cronbach's alpha coefficient of the C-NCAT was reported to be 0.946, and Cronbach's alpha coefficients of each dimension were reportedly 0.836–0.919, indicating good reliability of the C-NCAT [26]. The NCAT was translated, back-translated, and consulted by experts to adapt to Chinese culture. Therefore, the C-NCAT can effectively measure the level of nursing organizational culture perceived by Chinese nurses. And it has been widely used in China. In this study, the total Cronbach's alpha coefficient of the scale was 0.965, and Cronbach's alpha coefficients of each dimension ranged from 0.754 to 0.910.
4. The C-CLS [35]. The translation process of the C-CLS included five steps: translation, synthesis, back translation, expert committee review, and pre-test [35]. The scale comprises 15 items across five dimensions, namely, inspiring a shared vision (*n* = 3), modeling the way (*n* = 3), enabling others to act (*n* = 3), challenging the process (*n* = 3), and encouraging the heart (*n* = 3). The scale uses self-assessment and the 5-point Likert scoring method. The score indicates the frequency of the nurse's clinical leadership behavior, and higher scores indicate stronger clinical leadership. The content validity index was 1.0, indicating that the C-CLS had very satisfactory content validity [35]. The results of confirmatory factor analysis showed that the standardized factor loadings ranged from 0.5 to 0.95, and the model fitted well (chi-square-to-degrees of freedom ratio [*χ*^2^/df] = 2.413, root-mean-square error of approximation [RMSEA] = 0.079, Tucker–Lewis index [TLI] = 0.917, comparative fit index [CFI] = 0.941), indicating that the C-CLS had good construct validity [35]. The C-CLS reportedly had good reliability, with a total Cronbach's alpha coefficient of 0.942, Cronbach's alpha coefficients of 0.748–0.889 for each dimension, and a test–retest reliability coefficient of 0.892 [35]. In this study, the total Cronbach's alpha coefficient of the CLS scale was 0.960, and Cronbach's alpha coefficients of each dimension ranged from 0.844 to 0.904.

### 2.4. Data Collection

The researchers compiled questionnaires through the Questionnaire Star platform and conducted on-site questionnaire surveys during the collective meetings of each department. The researcher first explained the inclusion and exclusion criteria for the survey participants and informed them of the significance and purpose of the study, the filling method of the questionnaire, and the precautions that need to be taken. The research participants who met the inclusion criteria and signed the informed consent form scanned the QR code of the survey questionnaire to enter it and completed all the answers according to the instructions on the answer page. The questionnaire was filled out anonymously, emphasizing the confidentiality of data information. To ensure the completeness of the questionnaire, the researchers made all mandatory questions in the questionnaire. When submitting the questionnaire at the end, the respondents will be reminded that if they have not answered all the questions, they will not be able to submit the questionnaire and must complete all questions. To prevent duplicate questionnaire submissions, the researchers allowed only one questionnaire to be filled from one IP address. If the survey participants had any questions during the filling process, the researchers provided unified explanations and guidance on site in a timely manner.

### 2.5. Ethical Considerations

This study was approved by the Research Ethics Committee of Sir Run Run Shaw Hospital, Zhejiang University School of Medicine (approval number: 2023-920-01). During the investigation process, voluntary participation and anonymous filling were strictly followed, and the survey participants had the right to withdraw from the study midway. The collected data were only used for academic research, and the nurses' information was kept confidential.

### 2.6. Statistical Analysis

The survey data were directly imported into MS Excel software using the Questionnaire Star platform to establish a database. After two of the investigators of this study checked and cleaned the data and conducted logical checks, SPSS 25.0 and AMOS 24.0 software were used for statistical analysis. In this study, a two-sided test level of *α* = 0.05 and *p* < 0.05 were considered statistically significant. The frequency, composition ratio, mean, and standard deviation were reported in descriptive statistical analysis. The Pearson correlation analysis method was used to explore the correlation between variables. Multiple stepwise linear regression analysis was used to explore the influencing factors of staff nurse clinical leadership. AMOS24.0 software was used to construct a structural equation model of the relationships among ambidextrous leadership, nursing organizational culture, and staff nurse clinical leadership, and path analysis was performed on the association of the three variables. The bootstrap method was used to test the mediating effect of nursing organizational culture. The 95% confidence interval (CI) was calculated for 2000 bootstrap samples, and the values of the 95% CI that did not cross zero were considered to indicate a significant mediating effect [36]. Furthermore, this study used the following commonly used indicators to evaluate structural equation models: the *χ*^2^/df, RMSEA, normed fit index (NFI), goodness-of-fit index (GFI), CFI, incremental fit index (IFI), and TLI. When *χ*^2^/df was less than 5, and other indicators were greater than 0.90, the model fit was considered to be good and acceptable [37].

## 3. Results

This study included data from 472 nurses, mainly female (96.6%) and married nurses (60.6%), with most of them belonging to the age group of 30–39 years (46.4%), followed by 20–29 years (40.5%). Overall, 94.5% of nurses had a bachelor's degree, and the majority of them held the title of supervisor nurse (56.6%). The employment method was mainly based on employment contracts (60.2%). In terms of their work roles, the majority were general nurses (77.8%). Furthermore, in terms of nursing work experience, the majority of nurses had 1–5 years (33.3%) or 6–10 years (29.9%) of experience. The participants were predominantly from internal medicine and surgery departments, accounting for 27.8% and 35.8% of the nurses, respectively. In terms of participation in leadership training, the vast majority of nurses (81.8%) had never participated in leadership training. The score of head nurses' ambidextrous leadership perceived by staff nurses was 16.57 ± 4.99, with the lowest score for *active management by exception*. The average score of the nursing organizational culture was 3.40 ± 0.45, with the lowest score for *professional commitment*. The average score of staff nurse clinical leadership was 4.45 ± 0.49, with the lowest score for *inspiring a shared vision*. The specific scores of each variable in this study are shown in Table 1.

The results of the Pearson correlation analysis showed that head nurses' ambidextrous leadership perceived by staff nurses was positively correlated with staff nurse clinical leadership (*r* = 0.461, *p* < 0.01) and nursing organizational culture perceived by staff nurses (*r* = 0.641, *p* < 0.01). Nursing organizational culture was also positively correlated with staff nurse clinical leadership (*r* = 0.685, *p* < 0.01). The results of the correlation analysis are shown in Table 2.

The total score of staff nurse clinical leadership was considered as the dependent variable; demographic variables and statistically significant variables in correlation analysis results (ambidextrous leadership and nursing organizational culture) were considered as independent variables, and multiple categorical variables were set as dummy variables and entered into the regression model. The results revealed professional title, role at work, marital status, participation in leadership training, and nursing organizational culture as the main predictors of staff nurse clinical leadership, collectively accounting for 49.8% of the total variation (Table 3).

**Figure 1:**
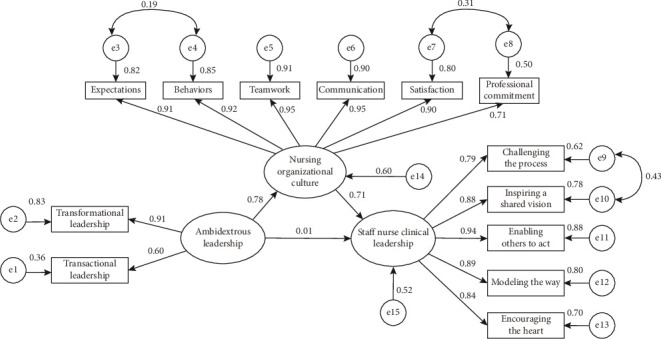
Structural equation model of ambidextrous leadership, nursing organizational culture, and clinical leadership.

We used AMOS to construct a structural equation model of ambidextrous leadership, nursing organizational culture, and staff nurse clinical leadership to examine the mediating role of nursing organizational culture. We used the maximum likelihood estimates method in the software to estimate the loading of each factor and adjusted the model based on the modification indicators to obtain a well-fitted structural equation model, as shown in Figure 1. The fitting index of the structural equation model was as follows: *χ*^2^/df = 3.325, RMSEA = 0.070, GFI = 0.942, NFI = 0.970, IFI = 979, TLI = 0.972, and CFI = 0.979.

The path analysis results of the structural equation model showed that ambidextrous leadership had a positive impact on nursing organizational culture (standardized coefficient *β* = 0.776, *p* < 0.001). The higher the level of head nurses' ambidextrous leadership perceived by staff nurses, the higher the level of nursing organizational culture perceived by staff nurses. Nursing organizational culture also had a positive impact on staff nurse clinical leadership (standardized coefficient *β* = 0.715, *p* < 0.001); the higher the level of nursing organizational culture perceived by staff nurses, the higher their level of clinical leadership. However, the predictive effect of ambidextrous leadership on staff nurse clinical leadership was not significant (standardized coefficient *β* = 0.007, *p* = 0.916 [> 0.05, not significant]), which suggests that the direct impact of ambidextrous leadership on staff nurse clinical leadership is not significant.

We further examined the mediating effect of nursing organizational culture using the bootstrap bias-corrected 95% CI estimation method. The results showed that the deviation CI of the indirect impact path of ambidextrous leadership on staff nurse clinical leadership was 0.454–0.692, and the CI did not include 0. This showed that the mediating effect of nursing organizational culture was significant. Conversely, the deviation CI of the direct impact path of ambidextrous leadership on staff nurse clinical leadership was −0.133–0.137, and the CI included 0. This indicated that there was no direct effect of ambidextrous leadership on staff nurse clinical leadership. Consequently, nursing organizational culture had a complete mediating effect between head nurses' ambidextrous leadership and staff nurse clinical leadership. As shown in Table 4, the direct effect of ambidextrous leadership on staff nurse clinical leadership was 0.007, and the indirect effect of ambidextrous leadership on staff nurse clinical leadership through nursing organizational culture was 0.776 × 0.715 = 0.555. The total effect was 0.555 + 0.007 = 0.562, and the mediating effect accounted for 98.8% of the total effect.

## 4. Discussion

The results of this study showed that the staff nurse clinical leadership score was 66.69 (7.42), which was at an upper-middle level. This was higher than the survey results of Zhou et al. [38] on domestic clinical nurses but lower than the research results of Boamah [39] on Canadian nurses. This showed that although the clinical leadership practice of domestic nurses is good, there is still room for improvement. The score of the *challenging the process* dimension in this study was relatively low, which was consistent with the results of Zhou et al. [38] and lower than the results of Patrick et al. [4]. This can possibly be explained by the notion that the Chinese have been influenced by Confucianism for a long time and are innately unwilling to change, take risks, and compete, whereas Western culture advocates change, activity, innovation, competition, risk-taking, and thinking out of the box when competing. This suggests that head nurses should cultivate nurses' competitive spirit and innovation awareness in their continuing education, create a good, pro-innovation atmosphere for nurses, and increase and strengthen their training in evidence-based nursing. The *inspiring a shared vision* dimension scored the lowest in this study, which is consistent with the research results of Zhou et al. [38] and Cai et al. [16]. This finding can be attributed to the possibility that different work content and time allocation of medical and nursing staff make it difficult for medical and nursing staff to communicate effectively; it may also be because cross-professional team collaboration requires decision-making power, and clinical nurses do not have decision-making authority; furthermore, medical and nursing staff often have different voices in decision-making. However, the *inspiring a shared vision* dimension was highly correlated with positive results [5]. This suggests that head nurses and medical department managers should further optimize and reform the hospital management structure and system and that they should strengthen the voice, increase autonomy, and increase the decision-making power of nurses, particularly clinical nurses, in hospital management and clinical nursing. In addition, in this study, we found that only 18.2% of nurses had participated in leadership training, and leadership training is an effective way to develop staff nurse clinical leadership [40]. Cziraki et al. [41] found that the support of head nurses can improve clinical nurses' motivation and willingness to practice clinical leadership. Therefore, besides promoting leadership training for nurses, head nurses should express their support for staff nurse clinical leadership practices.

The results of this study showed that head nurses' ambidextrous leadership perceived by staff nurses and staff nurse clinical leadership were positively correlated, which is consistent with the results of Cai et al. [16]. According to the social information processing theory, leaders and their leadership styles, as important sources of information for employees in the workplace, have an important impact on employee behavior. Previous research has shown that managers' long-term use of a single leadership style would negatively affect employees' enthusiasm for innovative behavior [42]. When managers flexibly use transformational leadership style and transactional leadership style, the simultaneous and synergistic use of these two leadership styles can promote employees' innovative behavior. Active learning, innovative thinking, and innovative behavior have an important positive effect on nurses' professional growth and critical thinking abilities. Nurses with strong professional abilities and critical thinking have sufficient ability to provide support and guidance to other colleagues, patients, and family members in clinical nursing work. They also have the courage to question the problems encountered in their work that others consider normal. At the same time, they will form a unique influence and appeal, which can unite their followers and inspire them to work toward a common vision by setting an example for themselves. This is an important attribute of staff nurse clinical leadership. This suggests that head nurses should change their perception that only transformational leadership style is good and instead encourage head nurses to adopt a dual leadership style.

The results of this study showed that nursing organizational culture was positively correlated with staff nurse clinical leadership. Nursing organizational culture refers to the common values, basic beliefs, and behavioral norms gradually inculcated in nurses by nursing organizations, which have inherent guiding, cohesive, motivating, affinity, and restraining effects. Schein's organizational culture theory states that organizational culture determines organizational values and the behavior of organizational members under these values [19]. Studies have shown that a positive organizational culture can support employee growth, promote effective coordination and cooperation among employees, and improve organizational efficiency [43]. It can also improve nurses' work enthusiasm, work autonomy, and self-efficacy [44]. Self-efficacy refers to the ability to successfully interact with the environment, have confidence in achieving specific results, and have the ability to execute tasks and achieve the desired outcomes. It affects nurses' motivation and feelings as well as their thinking and behavior [45]. Research has shown that work engagement has a positive impact on clinical leadership in staff nurses [46]. Therefore, the higher the level of nursing organizational culture perceived by nurses, the higher the level of nurses' work enthusiasm, work engagement, work autonomy, and self-efficacy; it also makes the nurses more likely to take the initiative to learn, actively improve their abilities, work hard, and have more motivation and confidence to influence and lead colleagues, patients, and their families. Furthermore, it encourages them to work together to provide patients with high-quality nursing care. Therefore, head nurses should focus on shaping a positive nursing organizational culture in their work, which can be achieved by (i) promoting professional skills training, (ii) supporting and helping clinical nurses with career planning and providing them resources and opportunities to enhance their professional commitment, (iii) providing humanistic care and creating a relaxed and pleasant working atmosphere to improve job satisfaction, (iv) improving nursing standards and clarifying job roles and responsibilities, and finally, and (v) cultivating team awareness, improving communication skills, and promoting teamwork.

The results of multiple stepwise linear regression analysis showed that professional title, role at work, marital status, participation in leadership training, and nursing organizational culture were the main predictors of staff nurse clinical leadership. At the same time, the results showed that head nurses' ambidextrous leadership did not enter the regression equation of staff nurse clinical leadership, indicating that it was not an independent predictor of nurses' clinical leadership. This can perhaps be attributed to the sample size not being large enough to verify the direct effect of head nurses' ambidextrous leadership on staff nurse clinical leadership. Furthermore, it may also be that the improvement of head nurses' ambidextrous leadership level perceived by nurses cannot directly lead to the improvement of staff nurse clinical leadership level but relatively indirectly promotes nurses to carry out more frequent clinical leadership practice. Combined with the analysis of the relationship between ambidextrous leadership and organizational culture, as well as the relationship between organizational culture and staff nurse clinical leadership, we believe that the improvement of head nurses' ambidextrous leadership level will increase nurses' work enthusiasm, promote their innovative behavior, provide more work and learning resources, and create a more positive and beneficial organizational culture for nurses to further promote the improvement of staff nurse clinical leadership.

This study found that nursing organizational culture had a complete mediating effect between head nurses' ambidextrous leadership and staff nurse clinical leadership, indicating that head nurses' ambidextrous leadership indirectly affects the level of staff nurse clinical leadership via the complete mediating effect of nursing organizational culture. Schein's organizational culture theory believes that the leadership style of leaders shapes the corresponding organization's culture, which in turn affects the attitudes and behaviors of organizational members [19]. The research results of Alfadhalah et al. showed that transformational leaders developed, shaped, and maintained an organizational culture that was conducive to organizational innovation and learning by creating and instilling values, beliefs, and concepts that they believed were necessary for and beneficial to the organization, thus having a positive impact on the quality of care provided by the organization [47]. A previous report has shown that higher levels of transactional leadership can enhance employees' work centrality and thus improve their innovative behavior; work centrality reflects goal-oriented values, which in turn encourages employees to actively think and seek innovation to achieve their goals and to change their working methods to improve work efficiency [48]. The ambidextrous leadership model can not only weaken the shortcomings associated with using only one of the two leadership styles and eliminate the contradictions between them but also bring into play the synergy between the two. Leaders who adopt ambidextrous leadership have the characteristics of idealized influence and charisma, which can create a learning space for employees, provide reasonable authorization, and form a positive organizational culture. Therefore, employees are more likely to respect and trust such leaders, imitate their leadership behaviors, make extra efforts, actively improve their comprehensive abilities like their professional skills and communication skills, and actively play the role of leaders at work.

## 5. Conclusion

In this study, we found that nursing organizational culture can independently predict the level of staff nurse clinical leadership. Although head nurses' ambidextrous leadership did not directly affect staff nurse clinical leadership, it indirectly affected staff nurse clinical leadership via the complete mediating role of nursing organizational culture. Nursing organizational culture was found to have a full mediating effect between head nurses' ambidextrous leadership and staff nurse clinical leadership, and consequently, improving the level of head nurses' ambidextrous leadership and nursing organizational culture is conducive to enhancing staff nurse clinical leadership. Therefore, senior managers should encourage head nurses to adopt an ambidextrous leadership style and attribute significance to the important role of nursing organizational culture. Head nurses should also improve various rules and regulations to improve the enthusiasm of nurses; furthermore, they should promote leadership training for nurses at different levels and carry out reasonable authorization to promote the improvement of the level of staff nurse clinical leadership. This study utilizes two scales developed by non-Chinese scholars. Although these scales effectively measure each variable, it would be beneficial for Chinese scholars to create Chinese indigenous measurement scales for each variable. This would help further validate the conclusions of this study.

## 6. Limitations

Due to time and energy constraints, this study only surveyed nurses from a tertiary-level general hospital in Hangzhou, Zhejiang Province. A convenience sampling method was used to select research participants. Consequently, this study's sample is insufficient to represent the national nursing staff. Moreover, this research institution is a hospital with a Magnet designation [49], and the nursing organizational culture of this hospital may be relatively high, which may have a particular impact on the research results. Future research should further expand the geographical scope and sample size of the survey and include hospitals of different levels to optimize the representativeness of the research sample.

## Figures and Tables

**Table 1:** Mean and SD of the variables.

Item	Maximum	Mean	SD
Idealized influence	5	4.35	0.69
Inspirational motivation	5	4.31	0.66
Contingent reward	5	4.01	0.80
Active management by exception	5	3.41	1.03
Ambidextrous leadership	25	16.57	4.99
Behaviors	4	3.52	0.47
Teamwork	4	3.49	0.45
Communication	4	3.48	0.48
Expectations	4	3.45	0.47
Satisfaction	4	3.39	0.51
Professional commitment	4	3.16	0.58
Nursing organizational culture	4	3.40	0.45
Modeling the way	5	4.50	0.51
Enabling others to act	5	4.48	0.51
Encouraging the heart	5	4.46	0.59
Challenging the process	5	4.44	0.55
Inspiring a shared vision	5	4.35	0.59
Staff nurse clinical leadership	5	4.45	0.49

**Table 2:** Correlations between ambidextrous leadership, nursing organizational culture, and clinical leadership (*N* = 472).

	1	2	3	4	5	6	7	8	9	10	11	12	13	14	15	16
1	1															
2	0.548^∗∗^	1														
3	0.810^∗∗^	0.928^∗∗^	1													
4	0.671^∗∗^	0.433^∗∗^	0.606^∗∗^	1												
5	0.684^∗∗^	0.417^∗∗^	0.598^∗∗^	0.867^∗∗^	1											
6	0.671^∗∗^	0.446^∗∗^	0.615^∗∗^	0.854^∗∗^	0.900^∗∗^	1										
7	0.647^∗∗^	0.429^∗∗^	0.593^∗∗^	0.855^∗∗^	0.855^∗∗^	0.907^∗∗^	1									
8	0.624^∗∗^	0.433^∗∗^	0.583^∗∗^	0.836^∗∗^	0.807^∗∗^	0.835^∗∗^	0.870^∗∗^	1								
9	0.514^∗∗^	0.402^∗∗^	0.516^∗∗^	0.674^∗∗^	0.592^∗∗^	0.681^∗∗^	0.671^∗∗^	0.732^∗∗^	1							
10	0.690^∗∗^	0.471^∗∗^	0.641^∗∗^	0.918^∗∗^	0.896^∗∗^	0.945^∗∗^	0.936^∗∗^	0.919^∗∗^	0.835^∗∗^	1						
11	0.401^∗∗^	0.248^∗∗^	0.352^∗∗^	0.554^∗∗^	0.526^∗∗^	0.534^∗∗^	0.530^∗∗^	0.518^∗∗^	0.441^∗∗^	0.566^∗∗^	1					
12	0.480^∗∗^	0.344^∗∗^	0.447^∗∗^	0.612^∗∗^	0.586^∗∗^	0.604^∗∗^	0.591^∗∗^	0.586^∗∗^	0.514^∗∗^	0.638^∗∗^	0.820^∗∗^	1				
13	0.467^∗∗^	0.335^∗∗^	0.440^∗∗^	0.642^∗∗^	0.609^∗∗^	0.657^∗∗^	0.652^∗∗^	0.605^∗∗^	0.512^∗∗^	0.671^∗∗^	0.743^∗∗^	0.836^∗∗^	1			
14	0.444^∗∗^	0.309^∗∗^	0.413^∗∗^	0.580^∗∗^	0.565^∗∗^	0.578^∗∗^	0.582^∗∗^	0.561^∗∗^	0.474^∗∗^	0.608^∗∗^	0.703^∗∗^	0.786^∗∗^	0.831^∗∗^	1		
15	0.424^∗∗^	0.332^∗∗^	0.419^∗∗^	0.567^∗∗^	0.549^∗∗^	0.576^∗∗^	0.581^∗∗^	0.537^∗∗^	0.470^∗∗^	0.600^∗∗^	0.638^∗∗^	0.713^∗∗^	0.776^∗∗^	0.779^∗∗^	1	
16	0.493^∗∗^	0.349^∗∗^	0.461^∗∗^	0.656^∗∗^	0.630^∗∗^	0.655^∗∗^	0.652^∗∗^	0.623^∗∗^	0.536^∗∗^	0.685^∗∗^	0.868^∗∗^	0.925^∗∗^	0.927^∗∗^	0.907^∗∗^	0.871^∗∗^	1

*Note:* 1, transformational leadership; 2, transactional leadership; 3, ambidextrous leadership; 4, expectations; 5, behaviors; 6, teamwork; 7, communication; 8, satisfaction; 9, professional commitment; 10, nursing organizational culture; 11, challenging the process; 12, inspiring a shared vision; 13, enabling others to act; 14, modeling the way; 15, encouraging the heart; 16, clinical leadership.

^∗∗^ *p* < 0.01.

**Table 3:** The multiple stepwise linear regression analysis of staff nurse clinical leadership (*N* = 472).

Independent variable	B	SE	Beta	*t*	*p*
Constant	1.91	0.125		15.22	< 0.001
Primary nurse	—	—	—	—	Reference
Supervisor nurse	0.077	0.036	0.077	2.146	0.032
General nurse	—	—	—	—	Reference
Education nurse	0.158	0.073	0.076	2.184	0.029
Nursing team leader	0.141	0.048	0.103	2.96	0.003
Single	—	—	—	—	Reference
Divorced	0.181	0.145	0.041	1.244	0.214
Participation in leadership training	0.068	0.044	0.053	1.533	0.126
Nursing organizational culture	0.719	0.037	0.649	19.45	< 0.001

*Note:R* ^2^ = 0.504, adjusted *R*^2^ = 0.498, *F* = 78.757, *p* < 0.001, and Debin–Watson value is 1.936.

**Table 4:** Results for the total, indirect, and direct effects of ambidextrous leadership on staff nurse clinical leadership with nursing organizational culture as a mediator (*N* = 472).

Model pathways	Estimate	SE	95% CI		*p*	Effect proportion (%)
			Lower	Upper		
Direct effect	0.007	0.069	−0.133	0.137	0.916	1.2
Indirect effect	0.555	0.060	0.454	0.692	0.001	98.8
Total effect	0.562	0.043	0.474	0.649	0.001	

Abbreviations: CI, confidence interval; SE, standard error.

## Data Availability

The data that support the findings of this study are available on request from the corresponding author. The data are not publicly available due to privacy or ethical restrictions.
